# Effects of sleep fragmentation and partial sleep restriction on heart rate variability during night

**DOI:** 10.1038/s41598-023-33013-5

**Published:** 2023-04-17

**Authors:** Julia Schlagintweit, Naima Laharnar, Martin Glos, Maria Zemann, Artem V. Demin, Katharina Lederer, Thomas Penzel, Ingo Fietze

**Affiliations:** 1 Interdisciplinary Center of Sleep Medicine, Charité – Universitätsmedizin Berlin, Corporate Member of Freie Universität Berlin and Humboldt-Universität zu Berlin, Charitéplatz 1, 10117 Berlin, Germany; 2 Institute of Biomedical Problems, Russian Academy of Science, 76a, Khoroshevskoe Shosse, Moscow, Russia 123007; 3 Advanced Sleep Research GmbH, Luisenstraße 54-55, 10117 Berlin, Germany; 4 The Fourth People’s Hospital of Guangyuan, Guangyuan, China

**Keywords:** Stress and resilience, Non-REM sleep, REM sleep, Sleep, Sleep deprivation

## Abstract

We developed a cross-over study design with two interventions in randomized order to compare the effects of sleep fragmentation and partial sleep restriction on cardiac autonomic tone. Twenty male subjects (40.6 ± 7.5 years old) underwent overnight polysomnography during 2 weeks, each week containing one undisturbed baseline night, one intervention night (either sleep restriction with 5 h of sleep or sleep fragmentation with awakening every hour) and two undisturbed recovery nights. Parameters of heart rate variability (HRV) were used to assess cardiac autonomic modulation during the nights. Sleep restriction showed significant higher heart rate (*p* = 0.018) and lower HRV-pNN50 (*p* = 0.012) during sleep stage N1 and lower HRV-SDNN (*p* = 0.009) during wakefulness compared to the respective baseline. For HR and SDNN there were recovery effects. There was no significant difference comparing fragmentation night and its baseline. Comparing both intervention nights, sleep restriction had lower HRV high frequency (HF) components in stage N1 (*p* = 0.018) and stage N2 (*p* = 0.012), lower HRV low frequency (LF) (*p* = 0.007) regarding the entire night and lower SDNN (*p* = 0.033) during WASO during sleep. Sleep restriction increases sympathetic tone and decreases vagal tone during night causing increased autonomic stress, while fragmented sleep does not affect cardiac autonomic parameters in our sample.

## Introduction

Sufficient sleep is necessary for mental and physical health, serving recovery and well-being. It increases daytime concentration, cognitive function and regulates emotions^1^. However, many people are affected by disturbed sleep. In today’s society various external interventions in sleep rhythm like shift work, night work, professional on-call service or stress have the consequence of shortened sleep and sleep deprivation, or disrupted sleep by frequent awakenings during night. This can negatively affect well-being and may even lead to physical and mental impairment. Sleep deprivation increases morbidity and mortality rates from ischemic heart disease, stroke and cancer^2,3^. Other widespread diseases like hypertension and diabetes are also more common in subjects with chronic sleep deprivation^4,5^. The results of our study may be clinically relevant for persons with periods of insufficient or inefficient sleep like astronauts. A further investigation is planned in space related isolation projects under extreme situations to adjust a space schedule. Several experimental studies have investigated the influence of different sleep disturbances such as sleep deprivation^6,7^ and sleep fragmentation^8^ on biological parameters. However, only few compared the effects of those different external sleep disturbances^1,9^. In our study we focused on a comparison between sleep restriction and sleep fragmentation and the effects on cardiac autonomic parameters. Subjective and objective sleep efficiency and percentual distribution of each sleep stage in our study was already represented in a previous publication and showed significant changes: Overall sleep efficiency, objectively measured, showed no significant differences between all nights together. Corrected pairwise comparisons showed slight differences, e.g. during recovery nights after fragmentation. More details can be found in Laharnar et al. Range of objective sleep efficiency was between 82.2 and 88.7%^1^. Therefore, we wanted to strengthen these results using cardiac autonomic parameters. The autonomic nervous system is the interaction between sympathetic and parasympathetic pathways to modulate parameters like blood pressure and heart rate (HR) and their reaction to internal or external stimuli^10^. Heart rate variability (HRV) can provide information about functioning of the autonomic nervous system and interaction of sympathetic and parasympathetic (vagal) pathways: decreased HRV reflects autonomic dysfunction^11^. Sympathetic activity is increased due to a “fight and flight” reaction. Here, it increases HR and decreases HRV. Vagal activity reflects a “rest and digest” function, HR decreases and HRV increases^12^. HRV includes parameters of a time domain and a frequency domain. Time domain parameters are amongst others SDNN (standard deviation of NN-intervals), pNN50 (percent of NN-intervals longer than 50 ms from previous NN-interval), RMSSD (root mean square of successive differences of NN-intervals) and SDSD (standard deviation of successive differences). SDNN is a global marker for total HRV, whereas pNN50 and RMSSD reflect vagal activity^11^. Frequency domain parameters of the HRV include the VLF-band (very low frequency power (0.0033–0.04 Hz)), LF-band (low frequency power (0.04–0.15 Hz)), and HF-band (high frequency power (0.15–0.04 Hz)). An index like LF/HF-ratio can give information about sympathovagal balance^11,13,14^. While the effects of parasympathetic or sympathetic activation on either, VLF or LF are still unclear, HF is mostly affected by vagal activity^13,15^. The modulation of LF is already for a long time subject of research. Older publications state that LF is mainly modulated by the sympathetic nervous system^13,16,17^, but during the last years, a great number of authors claimed that LF is not a marker of sympathetic activity^18,19^. Goldstein et al. demonstrated that LF is not a direct marker of sympathetic activity, but is related to baroreflex function^20^. Reyes del Paso et al. confirmed this and also showed that there are still aspects that indicate that LF is even mainly affected by parasympathetical innervation^21^. Summing up, there are still discussions on the simplistic use of LF and other parameters of frequency domain and the modulation of LF is still not clear yet^22^.

Duration of sleep and therefore, sleep efficiency (total sleep time divided by time in bed) influence parameters of cardiac autonomic nervous system regulation^23,24^. Only ten percent loss of sleep efficiency provoke higher heart rate, lower HF, higher LF and higher LF/HF-ratio, indicating a shift towards greater sympathetic modulation^24^. The autonomic nervous system and thus HRV is modulated by various factors. Boudreau et al.^24^ claimed that HRV depends on sleep stage: deeper sleep stages are associated with vagal activity, whereas REM-sleep (rapid-eye-movement) is associated with sympathetic activity. They also showed that HRV varies in circadian rhythm independent from breathing. HRV is also affected by internal factors like baroreflex sensitivity^19^ and unspecific factors like age^25,26^, gender^27^, and diseases like hypertension^28^ and depression^29^. Sen et al. suggested that decreased HRV could be a predictor of mortality: abnormal parameters of HRV correlate with high risk of death^30,31^. Increased nocturnal HRV could also be seen as a predictor of cardiovascular diseases in patients with diabetes mellitus type 2^32^. Parameters of HRV correlate also with cardiac events, death and cognitive function^33^.

In our study we used an experimental design to evaluate and compare two specific sleep interventions, sleep fragmentation and sleep restriction. The aim was to investigate how these two types of intervention affect the cardiac autonomic tone. We hypothesized: Sleep restriction has a greater effect on heart rate and its variability during the night than sleep fragmentation; and a night with sleep restriction or sleep fragmentation shows higher sympathetic activity during night (indicating increased autonomic stress) than a night with undisturbed sleep.

Therefore, we assessed heart rate and its variability as the common marker of sympathetic and vagal activity.

## Materials and methods

Recruitment of participants, study design and detailed procedures are described in Laharnar et al.^1^. Below is a summary of relevant details and new aspects regarding analysis.

### Participants and recruitment

Twenty healthy men with a habitual nocturnal sleep time of seven to eight hours (controlled by a one-week actigraphy prior to study begin) participated in the study. Women were not included due to limited resources^1^. The study was approved by the local ethics committee (EA1/006/16) of the Charité—Universitaetsmedizin Berlin, and patients gave their written informed consent. All experiments were performed in accordance with relevant guidelines and regulations. The calculation of sample size was based on a previous study with sleep restriction^6^. Studies have shown that HRV measurements are reproducible and stable, inferring that a small sample size is acceptable^34^. Patients were informed about the sleep interventions and the study procedure.

### Study procedure

Participants were asked to keep a regular sleep–wake cycle with a nocturnal sleep of 7 to 8 h prior to study begin and during the nights without recordings. They were asked to keep regular work habits during the entire study.

**Figure 1:**
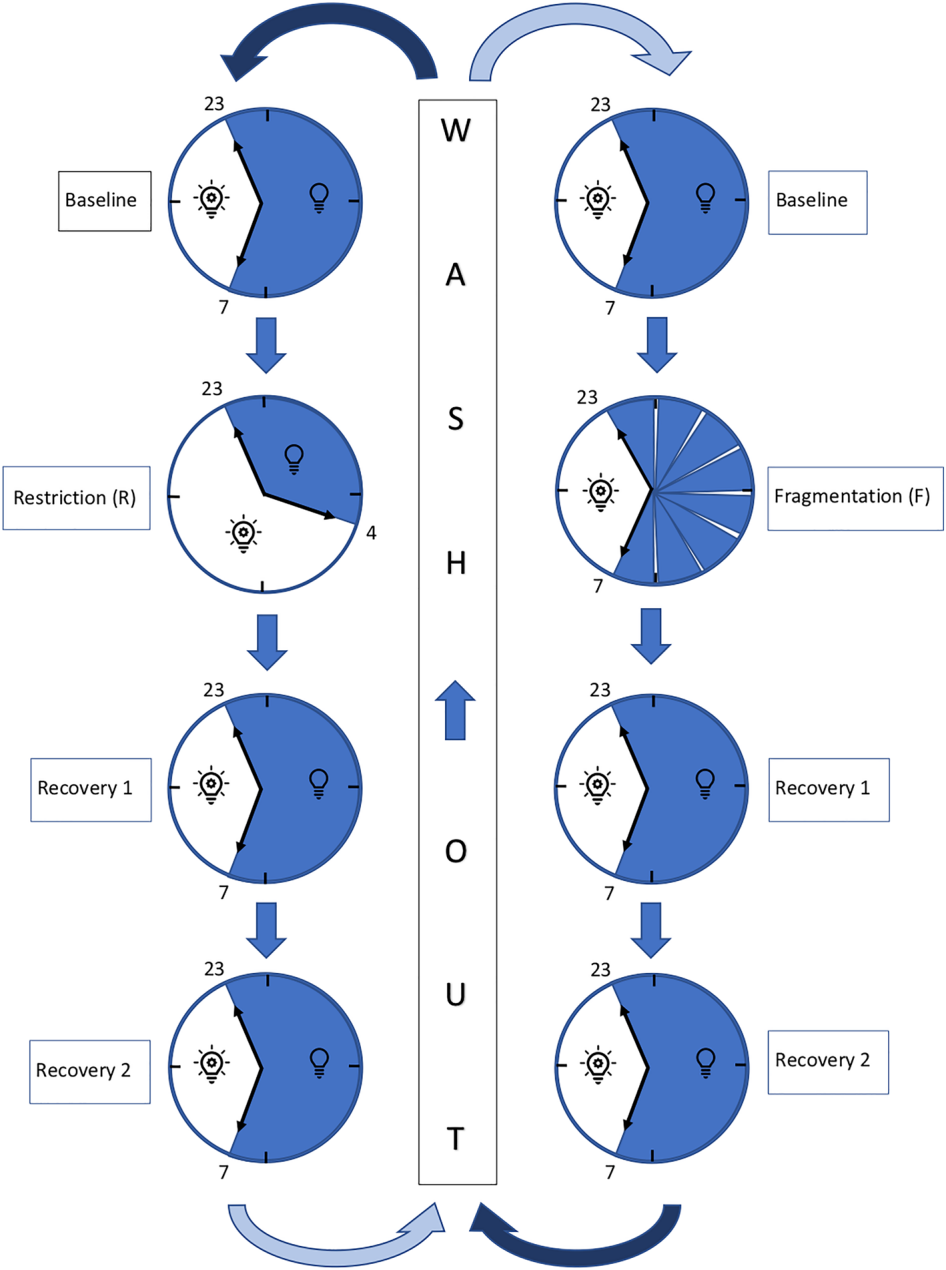
Study design with the interventions restriction (left side) and fragmentation (right side). A randomized cross-over-design was chosen with one group starting on the left side and one group starting on the right side. Light-on and light-off times are shown.

Each participant underwent 2 weeks of recordings, each week containing four nights (baseline, intervention, recovery, recovery) with an intermediate break of 11 days as a wash-out phase between both weeks. Sleep recordings were performed in a German Sleep Society (DGSM) board certified sleep lab. During baseline and recovery nights, participants had an undisturbed night with eight hours of sleep (light off: 11:00 pm, light on: 07:00 am). During the intervention night with sleep fragmentation, participants also slept for 8 h (11:00 pm until 7:00 am) but were woken up 7 times by turning light on every hour. Here, they filled out the 9-point Karolinska Sleepiness Scale (KSS), a short questionnaire on sleepiness. During the intervention night with sleep restriction, participants’ sleep was reduced to 5 h (11:00 pm until 04:00 am), after which there were woken up and spent additional 3 h awake in bed (see Fig. 1). Light-on and -out-hours in the evening, morning and every hour during fragmentation-night were performed and protocolled by trained sleep lab staff.

### Design and statistical analysis

This study was an experimental, randomized cross-over study, a within-subjects design with repeated measures. Each participant experienced both interventions: sleep fragmentation and sleep restriction (see Fig. 1). This design provides a better evaluation of within-person changes and each participant serves as his own control. Participants spent one baseline night, one intervention night (either fragmentation or restriction in a randomized order) and two subsequent recovery nights at the sleep laboratory. After a wash-out of eleven nights spent at home, the four laboratory nights were repeated with the other type of intervention. Below, the fragmentation night is called F, the restriction night R, the baseline night before fragmentation BF, baseline night before restriction BR, first recovery night after restriction R1R and second recovery night after restriction R2R.

Time series analysis of each laboratory night with subsequent 1-min epochs (from light-off-hour until light-on-hour) were performed. One minute was chosen as it is the shortest period to analyze LF-spectral band: 0.04 Hz corresponds to a period time of 25 s.

All HRV data analysis was performed according to the Task Force on HRV measurements^15^. The electrocardiogram was filtered and beat-to-beat time series of RR intervals were determined using an R-peak-detection algorithm. Heart rate was calculated. In time domain, SDNN, RMSSD, SDSD, and pNN50 were calculated. In frequency domain, based on Fourier spectral analysis, LF (0.04 to 0.15 Hz), HF (0.15 to 0.4 Hz) and LF/HF-ratio were calculated. Medians were calculated for each parameter and for every sleep stage. Additionally, medians for the first wake-period (between going to bed and falling asleep first time) were calculated for the analysis of HRV during sleep onset latency.

Two participants with more than 70% missing data during a night (due to missing or deficient electrocardiogram or electroencephalogram) were completely removed from the analysis, leaving 18 complete cases for analysis.

Data was checked for normal distribution with histograms and Q–Q-Diagrams. Kolmogorov–Smirnov and Shapiro–Wilk-Test were not used due to the small number of cases and to avoid multiple testing. As normal distribution could not be confirmed for all parameters and sleep stages, and because of the small case number, we applied non-parametric tests.

The four baseline and intervention nights (BR, R, BF, F) were compared using non-parametric Friedman-Test with adjusted *p*-level for multiple testing (Bonferroni-Correction). Using post-hoc Dunn–Bonferroni-Test, we compared pairwise both baseline-nights (BR–BF), the intervention with its corresponding baseline (BF–F and BR–R), and the interventions (R–F) in all Friedman-Tests with significant results. The analysis was repeated for each sleep stage. Significant results were displayed in boxplots, representing the median and interquartile range. In case of a significant difference between baseline and intervention, Friedman-test with adjusted *p*-level for multiple testing (Bonferroni-Correction) was used to find differences between recovery-nights and intervention/baseline-night. Also in case of a significant difference between baseline and intervention and between both interventions, HRV-parameters during sleep onset time was tested in this way. Friedman-Test was also used to compare autonomic tone of sleep stages within the single nights. Effect size r was calculated with values r < 0.3 as a weak, r = 0.3–0.5 as medium and r > 0.5 as a strong effect according to Cohen^35^. In order to ensure that there is no order effect, both baseline nights were compared using Wilcoxon-Test and both order groups were compared using Mann–Whitney-U-Test.

Data were statistically analyzed by using the software IBM SPSS Statistics, Version 25 (IBM, Corp., Armonk, NY). The same software was also used for charts. For all statistical tests, alpha level was set at p ≤ 0.05.

## Results

### Participants

Eighteen participants were included in our analysis. Mean (± SD) age of participants was 40.6 (± 7.5) years and mean Body Mass Index was 25.6 (± 2.3) kg/m^2^. Apnea–Hypopnea-Index was 1.52 ± 1.57 and habitual sleep duration was 7.6 ± 0.69 h per night. Two participants took antihistamine medication. Wash-out period between recording weeks consisted of 11.0 (± 0.0) nights.

### Preconditions for analysis

To ensure that there was no carry-over effect due to the order of the interventions and that the first night in each week can be set as baseline, a Wilcoxon-Test was used to compare both baselines and a Mann–Whitney-U-Test was used to compare both order groups (participants starting with intervention R vs. participants starting with intervention F). No significant difference between baseline nights or order of intervention groups was found. An order-effect and a carry-over-effect can be rejected.

All values and results of statistical analysis comparing nights can be read in Supplementary Table S1a–e.

### Results of comparing nights

**Figure 2:**
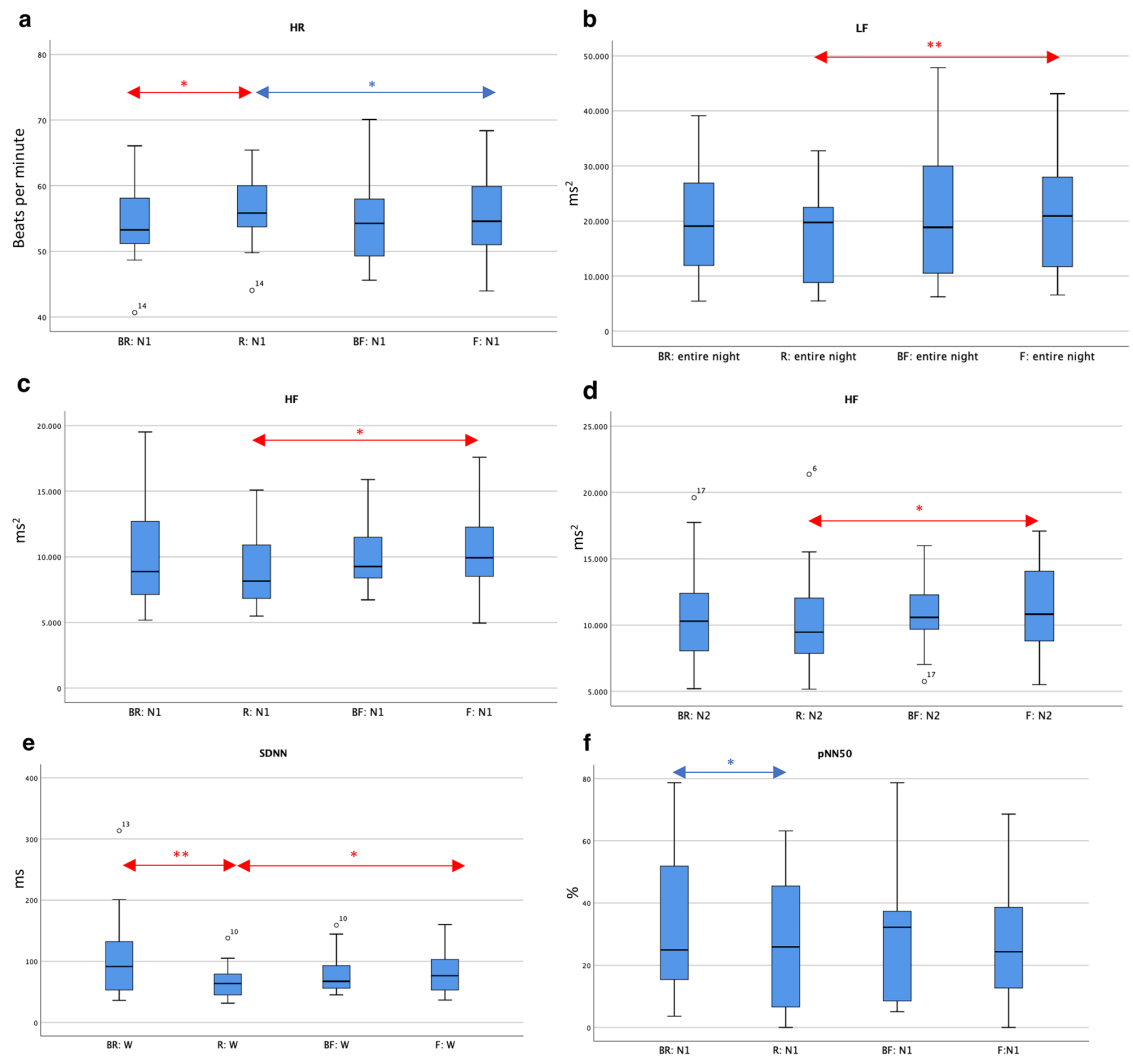
Presentation of significant differences in HR (heart rate) and HRV (heart rate variability) parameters between nights and in specific sleep stages. Presented are medians and interquartile range. The x-axis represents the nights (*BR* baseline night before restriction night, *R* restriction night, *BF* baseline night before fragmentation night, *F* fragmentation night) and the sleep stages (N1, N2 = Low sleep stages 1 and 2, W = wake-periods during night). The y-axis represents the value for the respective parameters (**a**) HR (heart rate) [beats per minute], (**b**) LF (low-frequency-band) [ms^2^], (**c**) HF in N1 (high-frequency-band) [ms^2^], (**d**) HF in N2 [ms^2^] (**e**) SDNN (standard deviation of NN-intervals) [ms], (**f**) pNN50 (proportion of number of interval differences of successive heart beats greater than 50 ms) [%]. Post-hoc Wilcoxon-Test was applied as a part of Friedman-Test, comparing the four nights. Red arrows show the significant differences with Bonferroni-correction for familywise errors, blue arrows show the significant differences without Bonferroni-correction. Significance levels are set at p < 0.05*, and p < 0.01**. Significant differences between an intervention night and the not-corresponding baseline night (e.g., F and BR) are not presented.

We found no significant difference using Friedman-Test between nights in parameters SDSD and RMSSD. All the significant differences comparing the nights and sleep stages are presented below and in Fig. 2.

#### Comparison of intervention and baseline nights

No significant differences between intervention night F and corresponding baseline night BF were found. All parameters were statistically identical in all sleep stages indicating that F has no or a very small impact on cardiac autonomic functioning. Comparing R with baseline BR, several significant differences regarding HR and HRV were found, suggesting a greater impact of R on the autonomic modulation of the heart (see Fig. 2). In BR, mean HR during N1 was 54.52 bpm (± 6.05), and in R, mean HR in N1 was 56.39 bpm (± 5.41), showing a significant higher HR during intervention night with a medium effect size of r = 0.30 (*p* = 0.018 *Bonferroni-corrected*). The same effect was found during WASO regarding SDNN, the global marker of HRV in time domain: R (65.25 ms ± 26.73) showed significant lower SDNN-values than BR (107.42 ms ± 71.44) with a medium effect size of r = 0.32 (*p* = 0.009 *Bonferroni-corrected*). Friedman-Test also showed that pNN50-distribution is not identical in N1-stages among the four nights (*p* = 0.027). Here, pairwise-comparisons also revealed a significant difference between R and BR with a weak effect size of r = 0.26 (*p* = 0.012*; Bonferroni-corrected: p* = 0.071). The intervention night R (26.08% ± 21.18) showed smaller values than BR (30.94% ± 21.59).

The higher HR, lower SDNN and lower pNN50 during the sleep restriction night compared to the corresponding baseline might reflect a shift towards increased sympathetic activity with less parasympathetic activity during a night with sleep restriction, especially for the light sleep stages and wake times.

#### Analyzation of recovery nights

Between BR and R, there were significant differences in N1 in heart rate, in N1 in pNN50 and during W in SDNN, so we examined if there was a recovery effect during the following two recovery nights.

In heart rate we found differences between R and R1R (*p* = 0.020, *Bonferroni-corrected*: 0.121) and between R and R2R (*p* = 0.010, *Bonferroni-corrected: p* = 0.059). In R1R mean HR in N1 was 54.03 bpm (± 5.65), in R2R 54.45 bpm (± 6.48), thus smaller than during N1 in R and not significant different to BR.

In SDNN, pairwise comparisons revealed a difference between R (65.25 ms (± 26.73)) and R1R (92.56 ms (± 35.04)) in WASO (*p* = 0.01, *Bonferroni-corrected: p* = 0.059).

Regarding HR and pNN50 in light sleep stage N1 respective WASO, there is a recovery effect within the first two nights following the intervention night towards the values of baseline night.

In N1, pNN50 did statistically not differ in BR and R vs R1R and R2R.

#### Comparison of fragmentation and restriction nights

During baseline nights, participants underwent full somnography and during the intervention-nights, only necessary sensors were applied, so the disturbance of sensors might have been less^1^. In order to further investigate a possible increase in sympathetic activity during R (excluding the influence of baseline), both intervention nights were directly compared with each other. Significant differences with Bonferroni-correction were found for the parameters HF, LF and SDNN (see Fig. 2). For HR a significant difference was found without applicating Bonferroni-correction: HR was higher during N1 in R (56.39 bpm ± 5.41) than during N1 in F (55.38 bpm ± 6.23) with a weak effect size of r = 0.22 (*p* = 0.028;* Bonferroni-corrected p* = 0.169).

SDNN was significant lower during WASO in R (65.25 ms ± 26.73) than during WASO in F (81.94 ms ± 33.12) with a weak effect size of r = 0.28 (*p* = 0.033, *Bonferroni-corrected*). This might indicate that sympathetic nervous systems activity was higher during sleep restriction. The effect could be confirmed with the parameter HF, as an indicator for vagal activity. HF was significant lower during R than during F in light sleep stages N1 (R: 9017.56 ms^2^ ± 2771.12; F: 10,329.83 ms^2^ ± 3181.16 with a *p* = 0.018 *(Bonferroni-corrected)*) with a medium effect of r = 0.30 and N2 (R: 10,296.94 ms^2^ ± 3858.07; F: 11,123.67 ms^2^ ± 3121.36 with a *p* = 0.012 (*Bonferroni-corrected*)) with a medium effect of r = 0.31. This indicates that fragmented sleep with a higher parasympathetic activity is less of a sleep disturbance than sleep restriction. Unexpectedly, LF was also significant lower in R (mean 17,663.83 ms^2^ ± 8660.89 than in F (20,850.89 ms^2^ ± 9779.16) regarding the entire night with a medium effect size of r = 0.33 and a *p* = 0.007 *(Bonferroni-corrected)*. LF often has an opposite behavior than HR^36^. Therefore, we calculated the ratio LF/HF, but could not find any significant difference.

#### Parameters of HRV during sleep onset time

No significant differences in HR, SDNN, pNN50, LF and HF were found during sleep onset time (wake time between going to bed and falling asleep for the first time). Consequently, it can be assumed, that the differences we found while compairing nights are only during night and not immediately before falling asleep.

### Comparison of inter-night and within-night changes

To be able to estimate the effect of sleep interventions, we analyzed the within-night-changes while comparing sleep stages of baseline nights using Friedman-Test and calculated effect sizes for this too. There were several significant differences in post-hoc pairwise comparisons with effect sizes up to r = 1.659 (N2-W) in HR, up to r = 0.67 (N3-W) in SDNN, up to r = 0.42 (W-N1) in pNN50, up to r = 0.67 (N1-N3) in LF and up to r = 0.628 (REM-N3) in HF.

Our data are suggesting that within night changes have thus a strong effect on HRV-parameters.

## Discussion

By using a cross-over within-subjects design with 20 young men, directly comparing two different sleep interventions, we suggest with our data that shortened sleep (sleep restriction) increases sympathetic tone and decreases vagal tone during night causing increased autonomic stress, while fragmented sleep does not affect cardiac autonomic parameters.

Laharnar et al. already showed, that there was no difference in objective sleep efficiency between the eight nights. Subjective sleep efficiency had lowest values after the intervention nights, but not significantly lower. They also claimed lower wake-times, less light sleep, less REM-sleep and more slow-wave-sleep during restriction night compared to fragmentation night. Regarding these, there was a recovery effect in in restriction week. Also PVT showed a recovery effect after restriction week. They concluded that restriction displayed a stronger sleep disturbance and a higher need for recovery than fragmentation^1^.

Our results point up these previous results on the level of autonomic parameters towards a higher sympathetic activity during R compared to F.

We could further show with our simplified hypotheses that light sleep stages (N1 and N2) were more affected, than deep sleep stages (N3) and REM sleep by sleep restriction.

Analyzation of HR and SDNN showed that already the on the intervention following night with undisturbed sleep shows similar heart rates and SDNN values than the baseline night does. Therefore, we assume the impact of sleep restriction to be short-termed.

HR was significant higher during light sleep stages of the restriction night compared to the corresponding baseline night which could show that this higher sympathetic activation is caused by the expectation of inefficient sleep. While participants were not blinded of the interventions, they were exactly informed of what to expect in both sleep intervention nights, sleep restriction as well as sleep fragmentation. Therefore, it is implausible that HR differences between the intervention nights were caused by expectations. To be on the safe side, we did the analysis , see “parameters of HRV during sleep onset time”,  which showed that there is no difference in HRV immediately before falling asleep, so that we can rule out that the nocturnal changes in cardiac autonomic tone are due to stress occurring before sleep.

However, it is noteworthy that participants did not complete the normal four to five complete cycles of sleep in the sleep restriction night due to the shortened sleep. Here, participants were woken up after only five hours (after light off time), three hours earlier than during baseline and fragmentation nights. This had especially an effect on REM and deep sleep stages. During a night with undisturbed sleep, the amount of deep sleep decreases with each complete sleep cycle while the amount of REM sleep increases. There are also physiological changes of HR during a night when comparing subsequent cycles: with each completed sleep cycle, RR-interval gets longer, thus HR shifts towards a slower beat as in light sleep stages^37^. Therefore, it is unclear whether HR in our study is only faster during the restriction night, because the participant does not complete later sleep cycles with smaller HR due to being woken up earlier. HR during the entire night also depends on how fast it increases after sleep onset.

Nevertheless, the increased HR during restriction night indicates less vagal influence and therefore, a lack of regeneration and recovery in this intervention night^1,37^. Lower vagal activity is also associated with increased stress^36^. As our participants were with a mean age of 41 years relatively young and sleep healthy, frequent but short awakenings during the night may have caused less stress than being awakened after only 5 h of sleep. It has to be mentioned that young subjects tolerate being awakened during night better than elder people due to physiological different sleep in old age and due to the increase of sleep disorders^38^. The HRV analysis of the frequency-domain parameter HF also confirmed our results and demonstrated with a decreased HF during light sleep stages of the sleep restriction night less vagal activity than during sleep fragmentation. However, the LF parameter is still unclear in literature. Provided that LF reflects both sympathetic and vagal changes, our LF results would confirm our previous results by demonstrating that autonomic balance during sleep fragmentation may be increased, including a shift to parasympathetic activity. We continued analyzing another index for sympathovagal balance: LF/HF-ratio. Other common normalized indexes like normalized LF (LFnu = LF/(LF + HF) and normalized HF (HFnu = HF7(LF + HF) are mathematically redundant in combination with LF/HF-ratio and thus predictable in both directions. While analyzing LF/HF-ratio, LFnu and HFnu are completely determined; therefore we refrained from analyzing these^39^. We did not analyze VLF, because it is not meaningful if using epochs ≤ 5 min (we chose 1 min epochs)^15^. We did not find any significant differences in the HRV parameter RMSSD parameter (also time-domain). However, Stein et al. mentioned that RMSSD and pNN50 represent a changing vagal activity, but are difficult to assess, because it is not distinguishable, whether respiratory sinus arrythmia or a scanning error provokes increased values^11^. Therefore, it is possible that there were no significant results in RMSSD, because this parameter reacts very sensitive to respiratory sinus arrythmia.

The HRV analysis of the time-domain showed that SDNN during WASO times was significant smaller during restriction night than fragmentation or corresponding baseline night. As SDNN reflects total HRV, the prior results can be confirmed: sleep restriction disturbs HRV more than fragmentation. HRV reflects the organism’s ability to adapt internal functions such as heart rate to environmental stimuli^40^. Sleep restriction may negatively affect this ability, making it harder for the body to adapt. That also complies with the fact that a low SDNN is associated with higher mortality risk after myocardial infarction^41^ and thus, is of clinical importance.

We conclude that sleep restriction, even if it is expected, seems to have more negative affect on the cardiac autonomic tone than an expected sleep fragmentation, and should therefore be avoided.

While there are a lack of studies investigating fragmented sleep and comparing it to restricted sleep, there are studies comparing sleep restriction to undisturbed sleep. Here, our results are in line with those studies. Castro-Diehl et al. also showed that patients had an increased HR and decreased HF (as a marker of parasympathetic nervous system) in the sleep restriction night compared to an undisturbed night. They concluded that shortened sleep causes a decrease in cardiac parasympathetic activity and/or an increase in sympathetic tone^23^. The literature has shown that sleep restriction decreases HRV (e.g. lower SDNN) and leads to an autonomic imbalance and increases HR^6,42–44^ as can also be confirmed with our results. Dettoni et al. and Bonnet et al. also found a higher sympathetic activation and a respectively lower vagal activation compared to undisturbed sleep: patients had higher LF- and lower HF-power during sleep restriction^7,44^.

One study compared HRV in restricted as well as fragmented sleep with contradictory results. They found increased HR and also increased HF during fragmentation compared to restriction night in healthy and relatively young men with a mean age of 29.0 ± 3.1 years. They concluded that fragmented sleep affects heart rate and its parameters more than restriction in younger subjects^45^. However, results are not comparable to our study as design, fragmentation condition, participant age differ. Questionable is also the increased HRV with the HR parameter as indication of the stronger effect of fragmentation.

There are certain limitations to the study. Probands were notably healthy and young respective middle aged, but they were chosen, because the experiment was planned to be repeated in space and astronauts are on average this age and of good health. Our results are not transferable to the large spectrum of patients.

We did not include women because other variables like the menstrual phase influence heart rate and its variability^46^. As our study design consisted of a cross-over design with repeated measures, we included a wash-out period of about 11 days between the two intervention weeks. Literature has shown that a wash-out period of already 1 week seems to be enough to avoid a carry-over effect due to the order of interventions^47–49^. We also checked for a possible carry-over effect. However, it cannot be completely ruled out that the order may not have had at least some effect on the night-to-night variability. As another precaution, we compared the intervention night with the preceding corresponding baseline night, which then served as a control.

While Lo et al.^50^ and Laharnar et al.^1^ showed that two nights of recovery are not sufficient for subjective recovery, we assume that two days of recovery plus eleven days of wash-out time are sufficient.

A possible limitation is also the kind of fragmentation: participants were woken up by switching on the light and had to complete a sleepiness scale. Then, the light was turned off again and participants were allowed to continue sleeping. Maybe this disturbance was not strong enough to interrupt the sleep so that we recorded greater results regarding sleep restriction.

In our study, patients were not blinded to the interventions. They were informed about the study procedure and what to expect each night. This already can cause stress and increase sympathetic tone. Nevertheless, information was given concerning both interventions. Additionally, there was no difference in HRV immediately before falling asleep, so that the concern, that the expectation of a sleep intervention caused more stress, could be refuted.

During baseline nights, more sensors were applied causing less comfort may increase sleep disturbance. Only one baseline was recorded, consequently, participants were not able to adjust to the new sleep environment. In the literature this can be found as a first-night effect^51^. This could explain, why the measurable effects of the interventions were relatively small. It may be that the baseline nights showed already higher sympathetic tone than a fully undisturbed night. In order to account for this, we also compared the intervention nights to each other without the influence of the baseline.

A quite large number of statistical tests were performed, so the differences may be related to type-I-error. The calculation of sample size was based on SDNN values obtained in a study with sleep restriction^6^ and resulted in a requirement of 17 subjects using a within-subject-design with repeated measures. Nevertheless our sample size, containing 18 full cases, was quite small and might not be representative especially for the entire society including elder people.

Our study was a prestudy for further experiments under isolation or in cosmos. Therefore, not only autonomic tone, but also the performance after a disturbed night must be investigated: the need for recovery, subjective well-being and analysis of the psychomotor vigilance test (PVT) can be read in Laharnar et al.^1^.

## Conclusion

Sleep restriction influences cardiac autonomic tone more than sleep fragmentation. There is a shift towards higher sympathetic activity and lower parasympathetic activity during restricted sleep, especially during light sleep stages. Here, HR increases and HRV decreases. This indicates that sleep restriction may cause more stress for the organism than a sleep fragmentated night. In general, our study showed that sleep interventions like fragmentation and restriction have an impact on parameters of the cardiac autonomic tone, especially during the light sleep stages. During REM and N3 sleep, the body is probably able to hold parameters stable: they do not change as much as they change anyway in a physiological manner. In a next step, the interventions may be modified, fragmentation may be increased. Also, recovery time should be investigated.

## Supplementary Information

Supplementary Table S1.

## Data Availability

The datasets generated during and/or analysed during the current study are available from the corresponding author on reasonable request.
