# Supplementary material for: Effects of sleep fragmentation and partial sleep restriction on heart rate variability during night
Source: Sci Rep. 2023 Apr 17;13:6202. doi: 10.1038/s41598-023-33013-5 (PMC10110519; doi:10.1038/s41598-023-33013-5)
Supplement: Supplementary file 1 — Supplementary Table S1. [file 41598_2023_33013_MOESM1_ESM.pdf]

**Effects of sleep fragmentation and partial sleep restriction on heart rate variability during night**

Schlagintweit Julia; Laharnar Naima; Glos Martin; Zemann Maria; Demin Artem V.; Lederer Katharina; Penzel Thomas; Fietze Ingo

Supplementary Table S1a-e: Heart rate and heart rate variability parameters and comparisons between nights

S1a: Heart Rate (HR)

| Parameter | Stage | Mean ± SD | | Friedman | Wilcoxon | Wilcoxon, Bonferroni-corrected | Effect size |
|---|---|---|---|---|---|---|---|
| **HR [beats per minute]** | N1 | BF | F | 0.014 | 0.699 | 1.000 | |
| | | 55.21 ±7.09 | 55.38±6.23 | | | | |
| | | BR | R | | 0.003 | 0.018 | 0.301 |
| | | 54.52±6.05 | 56.39±5.41 | | | | |
| | | R | F | | 0.028 | 0.169 | 0.223 |
| | | 56.39±5.410 | 55.38±6.23 | | | | |
| | N2 | BF | F | 0.228 | | | |
| | | 53.894±6.51 | 54.63 ±6.11 | | | | |
| | | BR | R | | | | |
| | | 53.70±5.84 | 55.54±6.28 | | | | |
| | | R | F | | | | |
| | | 55.54±6.28 | 54.63±6.11 | | | | |
| | N3 | BF | F | 0.108 | | | |
| | | 55.38±6.13 | 56.15±5.83 | | | | |
| | | BR | R | | | | |
| | | 55.53±6.22 | 56.83±6.33 | | | | |
| | | R | F | | | | |
| | | 56.83±6.33 | 56.15±5.83 | | | | |
| | REM | BF | F | 0.096 | | | |
| | | 55.96±7.26 | 56.84 ±6.75 | | | | |
| | | BR | R | | | | |
| | | 55.79±6.36 | 57.77±6.78 | | | | |
| | | R | F | | | | |
| | | 57.77±6.78 | 56.84 ±6.75 | | | | |
| | WASO | BF | F | 0.187 | | | |
| | | 61.03±7.35 | 61.19±6.47 | | | | |
| | | BR | R | | | | |
| | | 59.66±6.26 | 61.90 ±6.44 | | | | |
| | | R | F | | | | |
| | | 61.90±6.44 | 61.19±6.47 | | | | |
| | Entire night | BF | F | 0.182 | | | |
| | | 55.14±6.42 | 55.67±5.92 | | | | |
| | | BR | R | | | | |
| | | 54.97±6.10 | 56.47±6.24 | | | | |
| | | R | F | | | | |
| | | 56.47±6.24 | 55.67 ±5.92 | | | | |

S1b: Low Frequency Band (LF)

| Parameter | Stage | Mean ± SD | | Friedman | Wilcoxon | Wilcoxon, Bonferroni-corrected | Effect size |
|---|---|---|---|---|---|---|---|
| **LF [ms²]** | N1 | BF | F | 0.559 | | | |
| | | 30,849.56 ±18,437.68 | 28,343.89 ±13,607.99 | | | | |
| | | BR | R | | | | |
| | | 26,986.06 ±15,057.39 | 24,401.00 ±16,898.00 | | | | |
| | | R | F | | | | |
| | | 24,401.00 ±16,898.00 | 28,343.89 ±13,607.99 | | | | |
| | N2 | BF | F | 0.519 | | | |
| | | 19,763.00 ±11,403.22 | 19,970.78 ±9,315.69 | | | | |
| | | BR | R | | | | |
| | | 19,749.89 ±11,374.42 | 22,346.39 ±16,809.02 | | | | |
| | | R | F | | | | |
| | | 22,346.39 ±16,809.02 | 19,970.78 ±9,315.69 | | | | |
| | N3 | BF | F | 0.221 | | | |
| | | 12,753.22 ±7,480.26 | 14,705.06 ±9,418.12 | | | | |
| | | BR | R | | | | |
| | | 12,512.22 ±7,201.38 | 11,588.22 ±6,367.72 | | | | |
| | | R | F | | | | |
| | | 11,588.22 ±6,367.72 | 14,705.06 ±9,418.12 | | | | |
| | REM | BF | F | 0.133 | | | |
| | | 30,047.56 ±20,944.96 | 31,033.44 ±19,644.69 | | | | |
| | | BR | R | | | | |
| | | 26,507.50 ±18,269.69 | 27,545.33 ±20,186.97 | | | | |
| | | R | F | | | | |
| | | 27,545.33 ±20,186.97 | 31,033.44 ±19,644.69 | | | | |
| | WASO | BF | F | 0.352 | | | |
| | | 27,686.44 ±13,264.75 | 25,339.78 ±11,202.80 | | | | |
| | | BR | R | | | | |
| | | 23,877.39 ±13,872.92 | 23,227.17 ±13,898.55 | | | | |
| | | R | F | | | | |
| | | 23,227.17 ±13,898.55 | 25,339.78 ±11,202.80 | | | | |
| | Entire night | BF | F | 0.005 | 0.606 | 1.000 | |
| | | 20,556.56 ±11,791.69 | 20,850.89 ±9,779.16 | | | | |
| | | BR | R | | 0.197 | 1.000 | |
| | | 19,300.28 ±9,975.45 | 17,663.83 ±8,660.88 | | | | |
| | | R | F | | 0.001 | 0.007 | 0.327 |
| | | 17,663.83 ±8,660.88 | 20,850.89 ±9,779.16 | | | | |

S1c: High Frequency Band (HF)

| Parameter | Stage | Mean ± SD | | Friedman | Wilcoxon | Wilcoxon, Bonferroni-corrected | Effect size |
|---|---|---|---|---|---|---|---|
| **HF [ms²]** | N1 | BF | F | 0.028 | 0.302 | 1.000 | |
| | | 10,106.89 ±2,514.10 | 10,329.83 ±3,181.16 | | | | |
| | | BR | R | | 0.071 | 0.424 | |
| | | 10,1146.67 ±4,139.78 | 9,017.56 ±2,771.12 | | | | |
| | | R | F | | 0.003 | 0.018 | 0.301 |
| | | 9,017.56 ±2,771.12 | 10,329.83 ±3,181.16 | | | | |
| | N2 | BF | F | 0.013 | 0.439 | 1.000 | |
| | | 10,723.17 ±2,446.60 | 11,123.67 ±3,121.36 | | | | |
| | | BR | R | | 0.197 | 1.000 | |
| | | 11,150.22 ±3,924.60 | 10,296.94 ±3,858.07 | | | | |
| | | R | F | | 0.002 | 0.012 | 0.314 |
| | | 10,296.94 ±3,858.07 | 11,123.67 ±3,121.36 | | | | |
| | N3 | BF | F | 0.115 | | | |
| | | 12,273.89 ±4,202.50 | 12,707.89 ±4,892.20 | | | | |
| | | BR | R | | | | |
| | | 11,506.06 ±4,407.54 | 10,287.17 ±3,864.71 | | | | |
| | | R | F | | | | |
| | | 10,287.17 ±3,864.71 | 12,707.89 ±4,892.20 | | | | |
| | REM | BF | F | 0.072 | | | |
| | | 7,974.44 ±2,504.85 | 7,895.83 ±2,489.16 | | | | |
| | | BR | R | | | | |
| | | 7,684.00 ±2,009.08 | 6,880.22 ±1,678.46 | | | | |
| | | R | F | | | | |
| | | 6,880.22 ±1,678.46 | 7,895.83 ±2,489.16 | | | | |
| | WASO | BF | F | 0.086 | | | |
| | | 9,233.50 ±2,551.60 | 8,569.28 ±3,095.44 | | | | |
| | | BR | R | | | | |
| | | 8,752.33 ±3,290.30 | 9,187.72 ±3,236.92 | | | | |
| | | R | F | | | | |
| | | 9,187.72 ±3,236.92 | 8,569.28 ±3,095.44 | | | | |
| | Entire night | BF | F | 0.122 | | | |
| | | 9,930.89 ±2,145.52 | 10,459.94 ±2,777.89 | | | | |
| | | BR | R | | | | |
| | | 9,907.89 ±2,987.40 | 9,422.44 ±2,671.37 | | | | |
| | | R | F | | | | |
| | | 9,422.44 ±2,671.37 | 10,459.94 ±2,777.90 | | | | |

S1d: Standard Deviation of NN-Intervals (SDNN)

| Parameter | Stage | Mean ± SD | | Friedman | Wilcoxon | Wilcoxon, Bonferroni-corrected | Effect size |
|---|---|---|---|---|---|---|---|
| **SDNN [ms]** | N1 | BF<br>80.94 ±33.27 | F<br>75.58±37.03 | 0.359 | | | |
| | | BR<br>95.03±60.62 | R<br>78.92 ±33.21 | | | | |
| | | R<br>78.92±33.21 | F<br>75.58 ±37.03 | | | | |
| | N2 | BF<br>62.92±31.32 | F<br>63.94±34.08 | 0.796 | | | |
| | | BR<br>78.08 ±69.25 | R<br>66.14 ±38.02 | | | | |
| | | R<br>66.14 ±38.02 | F<br>63.94 ±34.08 | | | | |
| | N3 | BF<br>48.97 ±22.86 | F<br>66.75 ±72.62 | 0.618 | | | |
| | | BR<br>46.86 ±23.92 | R<br>49.42 ±25.64 | | | | |
| | | R<br>49.42 ±25.64 | F<br>66.75 ±72.62 | | | | |
| | REM | BF<br>71.42 ±33.16 | F<br>68.61 ±30.67 | 0.869 | | | |
| | | BR<br>83.58 ±68.13 | R<br>69.50±37.79 | | | | |
| | | R<br>69.50 ±37.79 | F<br>68.61 ±30.67 | | | | |
| | WASO | BF<br>79.97 ±33.97 | F<br>81.94 ±33.12 | 0.007 | 0.478 | 1.000 | |
| | | BR<br>107.42 ±71.44 | R<br>65.25 ±26.73 | | 0.002 | 0.009 | 0.321 |
| | | R<br>65.25 ±26.73 | F<br>81.94 ±33.12 | | 0.006 | 0.033 | 0.281 |
| | Entire night | BF<br>62.08 ±28.87 | F<br>62.47±31.61 | 0.539 | | | |
| | | BR<br>73.44±54.12 | R<br>59.78 ±29.79 | | | | |
| | | R<br>59.78 ±29.79 | F<br>62.47 ±31.61 | | | | |

S1e: Percent of NN-Intervals longer than 50 ms from previous NN-Interval

| Parameter | Stage | Mean ± SD | | Friedman | Wilcoxon | Wilcoxon, Bonferroni-corrected | Effect size |
|---|---|---|---|---|---|---|---|
| **pNN50 [%]** | N1 | BF | F | 0.027 | 0.107 | 0.639 | |
| | | 29.58 ±21.66 | 26.88 ±18.39 | | | | |
| | | BR | R | | 0.012 | 0.071 | 0.255 |
| | | 30.94 ±21.59 | 26.08 ±21.18 | | | | |
| | | R | F | | 0.439 | 1.000 | |
| | | 26.08 ±21.18 | 26.88 ±18.39 | | | | |
| | N2 | BF | F | 0.413 | | | |
| | | 29.88 ±24.23 | 27.57 ±22.81 | | | | |
| | | BR | R | | | | |
| | | 29.34 ±24.04 | 27.27 ±24.85 | | | | |
| | | R | F | | | | |
| | | 27.27 ±24.85 | 27.57 ±22.81 | | | | |
| | N3 | BF | F | 0.307 | | | |
| | | 28.26 ±25.54 | 24.10 ±23.13 | | | | |
| | | BR | R | | | | |
| | | 25.41 ±24.50 | 24.99 ±25.87 | | | | |
| | | R | F | | | | |
| | | 24.99 ±25.87 | 24.10 ±23.13 | | | | |
| | REM | BF | F | 0.060 | | | |
| | | 24.19 ±21.02 | 21.64 ±18.42 | | | | |
| | | BR | R | | | | |
| | | 24.47 ±22.57 | 20.06 ±21.05 | | | | |
| | | R | F | | | | |
| | | 20.06 ±21.05 | 21.64 ±18.42 | | | | |
| | WASO | BF | F | 0.820 | | | |
| | | 20.16 ±20.50 | 19.10 ±15.42 | | | | |
| | | BR | R | | | | |
| | | 24.82 ±20.96 | 20.08 ±19.19 | | | | |
| | | R | F | | | | |
| | | 20.08 ±19.19 | 19.10 ±15.42 | | | | |
| | Entire night | BF | F | 0.206 | | | |
| | | 26.79 ±23.26 | 24.68 ±21.63 | | | | |
| | | BR | R | | | | |
| | | 26.63 ±23.66 | 24.41 ±24.02 | | | | |
| | | R | F | | | | |
| | | 24.41 ±24.02 | 24.68 ±21.63 | | | | |

*Note: Only if Friedman-Test was significant with a p<0.05, post-hoc-test with pairwise comparisons (Wilcoxon-Test) were performed. Only if pairwise comparison showed a p<0.05, effect size was calculated.*
*Red indicates a significant difference between nights with Bonferroni-correction for familywise errors, blue indicates a significant difference between nights without Bonferroni-correction.*
*BF=Baseline-night before fragmentation, F=Fragmentation-night, BR=Baseline-night before restriction, R= Restriction night*
